# Ellis’s Medical Formulary

**Published:** 1854-02

**Authors:** 


*Ellis's Medical Formulary.* By R. P. Thomas, M. D. Blanchard & Lea, 1854.

"The elegant and judicious formation of prescriptions is one of the difficulties which the young practitioner in medicine is obliged to encounter." "To obviate in some measure, the inconvenience which the graduate at first experiences, the volume now offered to the public was undertaken and executed."—(From the author's preface.)

We invite the attention of the stripling in medicine to the following learned and erudite formula—page 96—Expectorants:

*Syrup of Squills.*

℞ Syrupi Scillæ f℥j.

*Signa*—Take a teaspoonful every three hours.

*Syrup of Garlic.*

℞ Syrupi Allii f℥j.

*Signa*—Give a teaspoonful to a child every three hours.

"This, like the syrup squills, is an excellent expectorant, being somewhat stimulant in its character." We advise those of our readers, if any such there be, who have not the knowledge and judgment to adapt their prescriptions to the condition of their patients to purchase the book; they will find it an indispensable volume; the scientific nature of their formulas giving them character with their druggists and patrons, with a *powerful* saving of brain labor.

For sale by Keen, Bro's & Co. J.

